# Mathematical modelling approach to understanding the effect of Mg-rich synthetic gypsum used as fertilizer on growth of *Hevea brasiliensis* in acid soils

**DOI:** 10.1371/journal.pone.0307476

**Published:** 2025-01-03

**Authors:** Fatai Arolu Ayanda, Mohd Firdaus Mohd Anuar, Susilawati Kashim, Oladosu Yusuff, Ibrahim Wasiu Arolu, Olabisi Fatimo Adekola, Adrian Crew

**Affiliations:** 1 School of Applied Sciences, University of West of England, Bristol, United Kingdom; 2 Department of Land Management, Faculty of Agriculture, Universiti Putra Malaysia (UPM), Serdang, Selangor, Malaysia; 3 Institute of Tropical Agriculture and Food Security (ITAFOS), Universiti Putra Malaysia (UPM), Serdang, Selangor, Malaysia; 4 Crop Science Department, Kaduna State University, Kaduna, Nigeria; 5 Department of Agronomy, University of Ilorin, Ilorin, Nigeria; Hainan University, CHINA

## Abstract

Knowledge of plant growth dynamics is essential where constraints such as COVID-19 lockdown restrictions have limited its field establishment. Thus, modeling can be used to predict plant performance where field planting/monitoring cannot be achieved. This study was conducted on the growth dynamics of rubber planted on two acid soils treated with either dolomitic limestone (GML), kieserite or Mg-rich synthetic gypsum (MRSG) to supply the Mg required by rubber seedlings. To understand the effect of applied treatments on the changes in rubber growth, data on plant height, stem diameter and biomass were regressed against months after transplanting (MAT) using the equation y = A/ (1+be^-ct^), and its derivative $\frac{dy}{dt}=\frac{Abce^{-ct}}{(1+be^{-ct)\;z}}$ was utilized for estimating the growth rate of the parameters. The dynamics in plant height, stem girth and plant biomass were modelled using an exponential function of y = Ae^bt^ and their rate of change was derived using dx/dy = Abe^bt^. The experiment indicated that the logistic growth curve model expressed as y = A/ (1+be^-ct^), closely described the growth in terms of each parameter against months after transplanting. A high probability level (a = 0.0001) was recorded in the model for all the treatments in the study. The growth of rubber seedlings in the glasshouse was improved by MRSG treatment in the two studied soils (Ultisol and Oxisol), giving comparable results to other Mg fertilizer treatments. The plant performed better on the Ultisol compared to the Oxisol. The results indicate the potential of using MRSG to replace conventional Mg-fertilizers to sustain rubber seedling growth.

## 1.0 Introduction

Advancement in the management of tropical acid soils for improved production of forage and plantation crops is made possible through liming to reduce soil acidity and the use of fertilizers to replenish lost nutrients [1, 2]. Nutrient management has been researched to be a major factor in rubber productivity, for example, Gohet *et al*. [3] reported that the influence of fertilizer application on latex yield ranged from + 5 to + 10%. Research shows that fertilization of mature rubber plantations is only profitable during periods of high Natural Rubber prices [4]. Although latex is not tapped during the early stage of rubber growth, optimal nutrition of the plant during this phase is imperative for the high yield and profitability of the trees during its matured stage. Therefore, management of the plantation at the immature phase should optimize nutrient management for improved tree growth to achieve quick latex harvesting. In the context of rubber cultivation in both conventional and non-traditional growing areas, soil nutrients and fertility rank as the major challenge during the early growth phase [5]. The use of adequate amounts of N fertilizer is fundamental for the optimal growth of rubber trees in all ecosystems, including acid soils. Similarly, fertilizers such as triple superphosphate, muriate of potash and kieserite, among others, are important sources of P, K and Mg fertilization in acid soils. Magnesium is an essential component of plant chlorophyll, therefore it serves a vital role in photosynthesis. The role of Mg in plants includes increasing water and nutrient uptake, thereby improving nutrient-use-efficiency and phosphate transport in the plant [6]. Magnesium is important in rubber plants and its deficiency has been associated with disruption of various metabolic activities in the plant. For example, interruption of protein synthesis and reduced starch accumulation has been recorded in Mg-deficient plants [7].

Aside from fertilizer application, the use of dolomitic limestone (GML) is a standard practice to improve productivity in acid soil [2]. In an experiment reported by Shamshuddin and Fauziah [8], on lime application to a rubber plantation intercropped with corn, it was found that rubber trees grew faster, and latex was tapped one year earlier compared to the control without detrimental impact on latex yield. The effect of GML in hastening the tapping time may be due to the function of Ca as an important macronutrient that is vital for cell wall formation, thereby improving tree growth. Ca is essential for activating cell division in meristematic tissue and helps in rapid root growth. In a trial on acid soil enrichment using NPK and Mg fertilizer, Timkhum *et al*. [9] noted that calcium was deficient in the plant alongside N and P. Shamshuddin *et al*. [1] reported it is an expensive practice to lime plantations due to unsustainable cost associated with liming large hectares of plantations. Also, rubber has moderate tolerance to soil acidity; however, growth is usually reduced under acid soil conditions [10]. GML contains a valuable amount of secondary nutrients (Ca and Mg), and when applied as Mg fertilizer, has been reported to limit soil acidity effect and improve crop growth on acid soil [2]. However, the slow reactivity of GML has made kieserite a preferable source of Mg, for example in Malaysia, where considerable imports are required [11]. The United Nations COMTRADE report [12] on international trade recorded Malaysia’s imports of fertilizers as US$2.01 Billion in 2022. Meanwhile, the global Covid-19 pandemic has led to an economic depression and decreased capital for purchases of fertilizers by plantation owners. Thus, the government spends a huge amount of allocation on input subsidies. This has made it important to evaluate the use of locally available resources as replacements for imported fertilizers in terms of performance and cost-effectiveness. An industrial residue, Mg-rich synthetic gypsum (MRSG), was reported to have characteristics that promote its use as an alternative to kieserite and GML as a source of Mg fertilizer and secondary nutrients [13]. The use of this material may reduce soil acidity conditions common across the tropical region of the world, where the rubber is largely cultivated, and supplies Ca; an essential macronutrient that is lacking in the common Malaysian formulation for immature rubber [14]. These, along with the projected low cost of MRSG, may indicate its importance as a source of secondary nutrients in rubber plantations. Meanwhile, the nature of MRSG as a locally sourced material may be pivotal in driving transformative climate actions through reduced transport miles of imported fertilizers and carbon footprint towards the COP28 objective. Some advocates have mentioned replacing some fertilizers as a strategy for decarbonizing the fertilizer industry, thus attempting to replace imported materials with local alternatives of near or equal efficiency could be vital in achieving these goals.

A study was conducted to determine the impact of applying MRSG on the growth of rubber plants in the immature phase. Due to the inaccessibility to conduct regular measurements or proceed to field planting as a result of covid-19 lockdown restrictions, a modelling approach was employed to comprehend the effect of treatments on the growth dynamics of rubber plants. Modelling can be described as a simplified description of a complex reality, while mathematical formalism is an important means of describing attributes of the modelized reality [15]. Thus, modelling can be utilized to translate the result of a scientific study into a mathematical expression, thereby offering a better explanation for the phenomenon. This study employs a modelling approach to understand rubber growth in acid soils under different fertilizer sources in a glasshouse environment.

## 2.0 Materials and methods

### 2.1 Planting material and experimental design

In this experiment, three-month-old rubber seedlings (RRIM 2001) of the same height were purchased from the Rubber Industry Smallholders Development Authority (RISDA), Kg Awah, Pahang, Malaysia.

The soils used in the glasshouse experiment were collected from Universiti Putra Malaysia (UPM) Farm, Puchong (02.5858 N, 101.3847 E) and the Rubber Industry Smallholders Development Authority (RISDA) plantation in Kg Awah, Pahang, Malaysia (03.27362 N, 102.58044 E). The necessary permits and approval for field access and soil sampling were obtained from relevant authorities i.e. the UPM Research Management Centre (RMC) and RISDA, an agency under the Ministry of Rural and Regional Development in Malaysia. The sampling sites for this study were selected according to their soil pH and mineralogical characteristics, which are important in soil-crop productivity. The soil collected from UPM Farm and RISDA plantation were identified as Bungor and Segamat Series, respectively, using the guidelines established by the Malaysian Department of Agriculture [16]. Following the criteria established for Soil Taxonomy [17], the Bungor soil was classified as belonging to the clayey, kaolinitic, isohyperthermic family of Typic Paleudults, while the Segamat soil was identified as clayey, oxidic, isohypertermic family of Haplic Acrothox. According to USDA classification [17], the two soils are classified as Ultisols and Oxisols. These soils represent the largest block of tropical marginal lands and are extensively used for agricultural cultivation in the tropics.

The study was carried out in a glasshouse at the Universiti Putra Malaysia (UPM) Farm (2°58.59 N;101°38.47 E), Serdang, Selangor, Malaysia. The site has a humid tropical condition with an average rainfall of about 2700 mm and a mean temperature of 25.3°C per annum [18]. The relative humidity was estimated to range between 80% and 90%. The experiment was laid out as a two factorial Randomized Complete Block Design (RCBD) with five replications. The experimental unit (polybag) comprised individual rubber seedlings. The treatments were calculated on the basis of 20 kg soil. The amount of soil used to grow the rubber seedlings was one that would allow sufficient growth of the rubber seedling for the 1-year duration of the study. This method was used by a previous study on rubber seedling growth in the glasshouse for the duration of 9 months and above [19]. The treatments were: T1- NPK without a source of magnesium; T2—NPK + GML at standard rate; T3—NPK + Kieserite at standard rate; T4—NPK + MRSG at the recommended rate; T5—NPK + MRSG at double the recommended rate. The soil in the polybag was treated with different Mg fertilizer at three months interval (2, 5, 8, and 11 months) for a period of 12 months.

### 2.2 Fertilizer application

The rubber seedlings in the glasshouse were fertilized following the recommendations of the Rubber Industry Smallholder Development Agency (RISDA). For the duration of the one-year study in the glasshouse, the fertilization schedule was applied at 2, 5, 8 and 11 months at the rate of 75, 116, 136 and 156 g, respectively. The fertilizer recommendation for rubber nursery was formulated based on RISDA 1 fertilizer grade 10.7 N: 16.6 P: 9.5K: 2.4 Mg; however, straight fertilizers were used in this study. This study used urea, Triple superphosphate and Muriate of Potash as NPK fertilisers. Table 1 contains information the rate of treatment applied to rubber seedlings in the glasshouse. Kieserite and GML were included as treatments to compare their results with those of the MRSG, while MRSG at twice the recommended rate was included as treatment to understand the effect of excess MRSG on plant and soil properties on N: P_2_O_5_:K_2_O:MgO.

**Table 1:** Details of the treatments used in the study.

Treatment Identifier	Rate of Application
T1	NPKMg (using RISDA 1 fertilizer compound grade 10.7:16.6:9.5:2.4) = 75.0g/polybag (8.03g N: 5.48g P:5.91g K:1.08g Mg)
T2	NPK + GML (100% equivalent amount to the recommended Mg rate of RISDA 1)
T3	NPK + Kieserite (100% equivalent amount to the recommended Mg rate of RISDA 1)
T4	NPK + MRSG (100% equivalent amount to the recommended Mg rate of RISDA 1)
T5	NPK + MRSG (200% equivalent amount to the recommended Mg rate of RISDA 1)

### 2.3 Crop management

The rubber seedlings were manually irrigated daily for all treatments in the glasshouse study. The rubber seedlings growing in the glasshouse were treated with insecticide (LOGOR) and fungicide (KENCOZEB). The insecticide and fungicide were applied at 25 g and 10 mL in 10 L water and sprayed once every fortnight to prevent whiteflies in the glasshouse and fungi disease of rubber.

### 2.4 Measurement of growth parameters

The growth performance of rubber seedlings in this study was evaluated at three-month intervals to allow a sufficient period for noticeable differences in plant growth parameters due to the effect of different treatments. The parameters determined include height, girth, leaf area, plant biomass, and chlorophyll content. The details for evaluating the growth parameter are presented in Table 2.

**Table 2:** The method for evaluation of growth parameters.

Growth Analysis	Details
**Plant height**	The trait was measured on the stem at 10cm from the soil surface up to the shoot tip using a measuring tape. A permanent marker was used to demarcate the area on the stem which is 10 cm above the soil surface and subsequent reading was taken on the marked area.
**Plant girth**	The girth size was measured on the marked area at 10 cm from the soil level using a digital vernier calliper.
**Dry weight**	At harvest, the plant was separated into different parts i.e. leaves, stems and roots. The roots growing in the soil inside the polythene bag were carefully separated from the extended woody stem. The roots were immediately rinsed in water to remove the soil and soaked in water to retain the freshness. Before this, the separated plant parts were placed on a weighing balance to record the fresh weight of the plant parts

### 2.5 Statistical analyses

SAS version 9.3 software (SAS, Cary, USA) was used to analyse the data. The variance of the effect of the treatment on all the traits was analysed, while Tukey’s studentized range (HSD) was used to compare means at a significant level of p≤ 0.05. Changes in the height, stem girth and plant biomass of *hevea brasiliensis* plants were recorded every 3 months from the first month after transplanting. The measured heights of plant growth at selected time periods were visualized using plots. and the numerical modelling was done using the Gauss-Newton methods, and the non-linear procedure in the SAS package. The values of predicted data were then recorded.

### 2.6 Mathematical model in computing plant growth

The computation of crop growth rate (*GR*) and relative growth rate (*RGR*) in terms of height, girth per plant and total dry weight using the mean method is shown in Eqs 1 and 2 [20, 21]

$$GR=\;\frac{\left(G_{2}-G_{1}\right)}{\left(t_{2}-t_{1}\right)}$$ (1)

Where *G*_1_ and *G*_2_ are changes in plant height, stem girth, and biomass at time *t*_1_ and *t*_2_ respectively and was determined based on Eq 3. *GR* represents the plant height growth rate (*PGR*_*H*_), stem girth growth rate (*PGR*_*G*_), and biomass growth rate (*PGR*_*G*_) at times *t*_2_ and *t*_1_, and was determined based on Eq 4.

$$RGR=\frac{\left(lmW_{2}-lmW_{1}\right)}{\left(t_{2}-t_{1}\right)}$$ (2)

Where, *RGR* relates to relative height growth rate of the stem (*RGR*_*H*_), relative girth growth rate (*RGR*_*G*_)and relative biomass growth rate (*PGR*_*B*_). In this study, the unit is cm/month/plant or g/month/plant for biomass, and the unit for RGR_H/G is_ cm^-1^ month^-1^. The area of the ground area was not considered in the computation of plant growth rate.

#### 2.6.1 Growth computation model

Measurement of change in plant height, stem girth, and biomass were collected on-field to examine the difference in plant growth and development. Plant height (cm), stem girth and dry weight of each treatment regressed against month after transplanting to determine the coefficients of the growth model Eq 3.

$$y=\frac{A}{1+be^{-ct}}$$ (3)

[22]

Where y = respective plant parameters i.e. plant height, stem girth and plant biomass (dry weight) and t = months after transplanting. The A, b and c are regression constants (second-degree polynomial constants. The A describes asymptotic level of each parameter, with an initial value of A/(1+b). The PROC NLIN (METHOD = DUD) of the SAS package was used for model development. The above function is commonly used for describing organism or organ growth over time [23]. This function could also be used to describe biological and physical growth processes. For example, the description of leaf growth of *Scheifieara arboricola* grown in extremely high salinity conditions [24]. In this study, height, girth and total plant biomass (dry weight) were selected as plant physical parameters or variables and were used to compute GR in Eq 1. Height, girth and total plant biomass (dry weight) growth rates were computed through regression of the plant parameters using an exponential function (*e)* raised to the power of quadratic function.

#### 2.6.2 Growth rates

By taking the derivatives of Eq 3, the growth rates of plant height, girth, and biomass (dry weight) were computed using Eq 4 as follows:

$$\frac{dy}{dt}=\frac{Abce^{-ct}}{(1+be^{-ct)\;z}}$$ (4)

Thus, the respective growth rates were abbreviated as GGR for Girth growth rate (cm month^-1^); HGR for Height growth rate (cm month^-1^); and BGR for Biomass growth rate (cm month^-1^)

#### 2.6.3 Relative growth rate

This is defined as the ratio of plant growth rate (GGR/HGR/BGR) to the current (instantaneous) GGR/HGR/BGR at the respective growth rate. The relative growth rate (RGGR/RHGR/RBGR) was calculated as follows.

$$\text{RGGR}/\text{RHGR}/\text{BGGR}=\frac{1}{HGR/GGR/BGR}.\frac{d(GGR/HGR/BGR}{dt}$$ (5)

[20]

where HGR/GGR/BGR is growth rate at time t (months after transplanting), the value of growth rate was obtained from Eq 1, and d(GGR/HGR)/dt was computed from Eq (2).

## 3.0 Results and discussions

**Fig 1:**
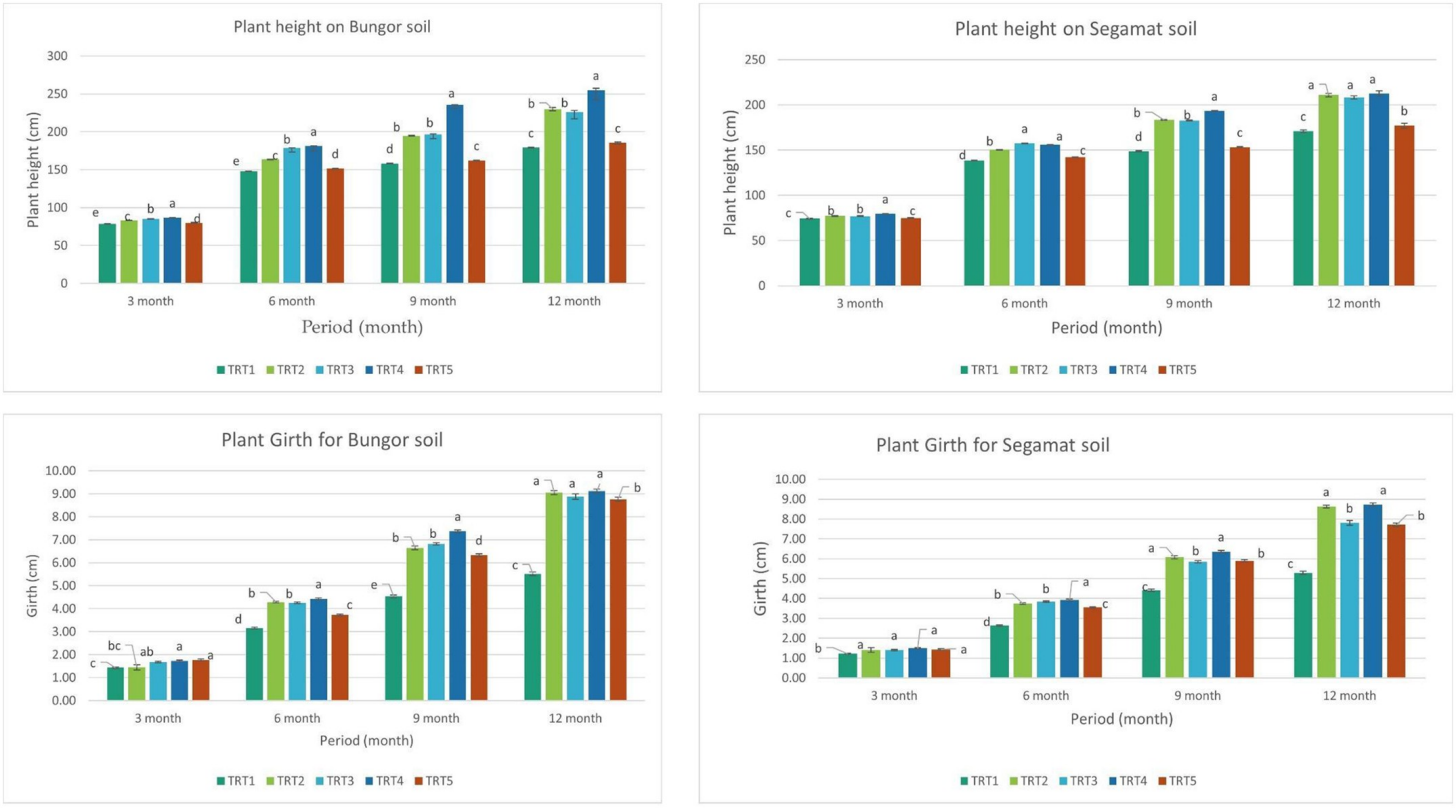
Height and girth of rubber seedlings growing in the Ultisol and Oxisol.

The effect of treatment was significant (p≤0.001) for all measured plant growth traits. The addition of MRSG improved the growth of rubber seedlings (through measurement of plant height, girth, and plant biomass) in the two experimented soils (Ultisol and Oxisol) This result is comparable to other sources of Mg fertilizer. Significant differences were also in the plant growth traits measured across the two different soil series (S). Height, girth and biomass showed significant effects among treatment applications and soil series (p ≤ 0.05). Meanwhile, growth responses showed significant differences in the interaction effect. The result indicated that the height increment of rubber seedlings was significantly influenced by MRSG starting from 3 months, and this trend continued until the study was concluded. The MRSG treatment resulted in the increased height of rubber seedlings (Fig 1) compared to other treatments that received equivalent amounts of Mg using different fertilizer sources, i.e. GML and Kieserite (T2 and T3) and MRSG at twice the recommended rate (T5).

The rubber seedlings performed better (p>0.05) in terms of height on the Ultisol belonging to the Bungor series compared to the Oxisol (Segamat series). The result also indicated an interaction effect of different soil types with different Mg fertilizers applied as treatment. It is known that Mg content in tropical soil is usually low and requires supplementation through the use of fertilizer [1]. Magnesium is an important element that affects chlorophyll formation and photosynthetic efficiency of plants i.e. the primary source through which plants derive their nutrient. Thus, it can be said that lower Mg will lead to lower photosynthesis and reduced nutrient synthesis in the plant, whereas its availability through Mg fertilizer application via different sources such as kieserite, GML and MRSG will lead to plant growth improvement. The significant influence of magnesium fertilizer on seedling height was consistent with previous experiments by Ayanda *et al*. (2020) [25] and Azham (2003) [26], where oil palms treated with magnesium fertilizers from different sources performed better than untreated palms. William *et al*. (2000) [27] observed a 15–20% reduction in the height of *Pinnus radiata* due to magnesium deficiency. Within the MRSG level, there was decreased height in MRSG treatment at double the recommended rate. This was attributed to the toxicity effect resulting from excess nutrients, which may lead to an imbalance in the plant system.

The rubber seedlings treated with Mg fertilizer, including MRSG, gave higher girth compared to control, whereas it was observed that there is no difference in girth of rubber seedlings amongst Mg fertilizer treatments. It was believed that the addition of magnesium fertilizer increased the rate of photosynthesis in Mg-treated seedlings which resulted in a higher growth rate of these seedlings. Ayanda [28] reported that the addition of MRSG as magnesium fertilizer increased the girth of oil palm seedlings planted on acidic Ultisol of Jempol series in Malaysia. Similarly, the `observation agreed with the previous finding reported by Goh et al. [29], and Than *et al*. [30], in separate studies on acid soils in Malaysia and Myanmar. The researchers both reported that the application of magnesium resulted in increased oil palm seedling growth on nutrient-deficient acid soils in their respective studies. The result of the mean comparison shows that girth size is not improved by MRSG treatment applied at twice the recommended Mg rate. Applying MRSG at double the recommended rate gave a significantly lower stem girth compared to other Mg treatments. This might be due to the effect of excess calcium present in the treatment. Paramananthan [31] reported that antagonistic reactions may occur due to a large amount of Ca, thereby inhibiting the uptake of other cations such as Mg. Thus, a lower photosynthesis rate due to inhibition of Mg by excess Ca in MRSG at double the recommended rate might have resulted in the lower stem girth recorded in this treatment.

The growing rubber plant performed better (p≤0.05) in terms of girth on the Ultisol belonging to the Bungor series compared to the Oxisol (Segamat) (Table 3). The chemical properties differences of both soil series might have caused the different effects of utilization and mechanism of the treatments in both soil series on height and girth increment.

**Table 3:** Effect of treatment on plant growth in the two studied soil.

	**Soil**	**Height**	**Girth**	**SFW**	**SDW**	**RFW**	**RDW**	**TFW**	**TDW**
At 3-month	Bungor	82.72a	1.61a	61.25a	24.71a	31.49a	11.97a	92.74a	36.69a
	Segamat	76.62b	1.39b	51.00b	20.22b	28.45b	10.82b	79.45b	31.05b
	MSD	0.55	0.06	1.09	0.56	0.51	0.19	1.2	0.59
At 6-month	Bungor	164.55a	3.97a	115.46a	50.64a	76.31a	26.95a	195.79a	94.84
	Segamat	148.81b	3.54b	90.60b	39.73b	60.39b	21.57b	150.98b	74.39
	MSD	0.5	0.03	2.4	1.06	1.83	0.71	2.83	1.49
At 9-month	Bungor	189.44a	6.34a	245.43a	111.61a	132.01	48.62a	377.44a	159.93a
	Segamat	172.27b	5.78b	168.19b	77.64b	90.68	33.70b	258.87b	109.67b
	MSD	0.64	0.03	1	2.35	0.88	0.53	1.37	0.58
At 12-month	Bungor	214.98a	8.27a	286.16	117.79a	151.08a	56.16a	437.24	183.48a
	Segamat	195.97b	7.63b	195.87	80.35b	107.95b	40.13b	303.95	127.55b
	MSD	2.54	0.11	4.07	1.38	4.04	1.5	6.31	2.65

Means followed by the same letter for each soil series within same column are not significantly different at p>0.05. SFW- shoot fresh weight, SDW-shoot dry weight, RFW- root fresh weight, RDW root dry weight, TFW- Total fresh weight, TDW-Total dry weight, MSD- Minimum Significant Difference

### 3.1 Rubber growth model

**Fig 2:**
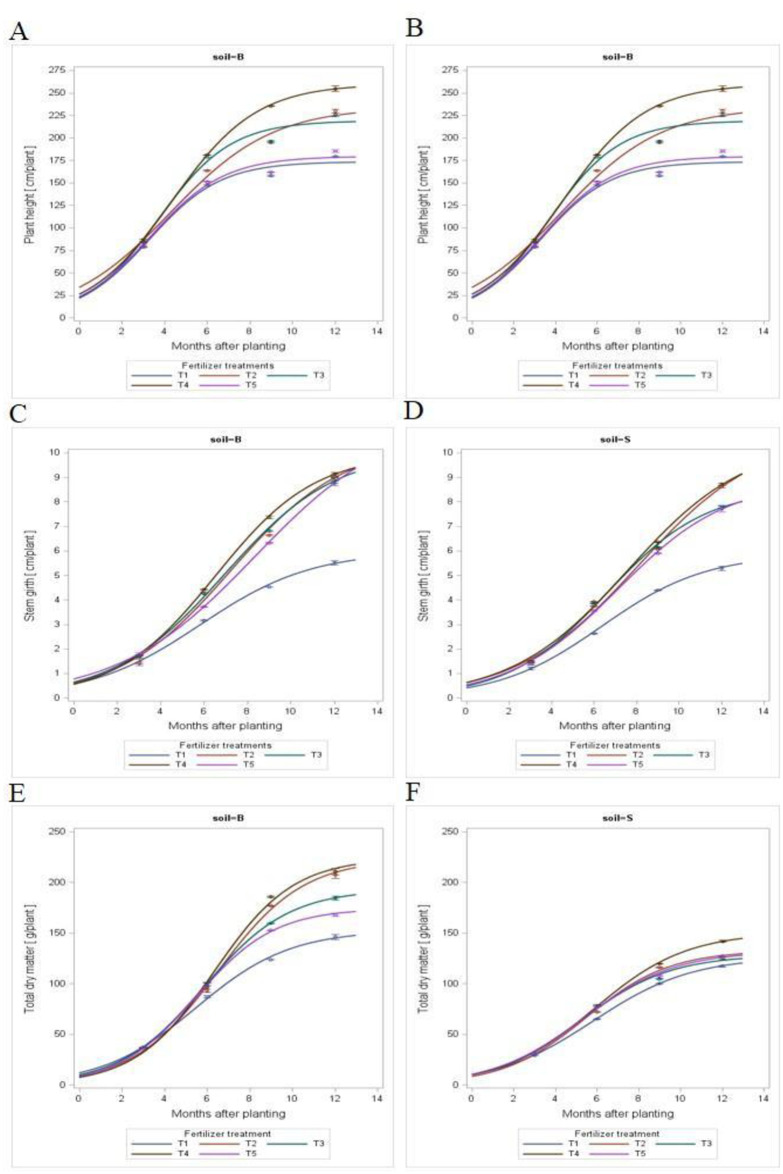
Plant height, stem girth and biomass on the two studied soils (B-Ultisol and S-Oxisol) versus months after planting expressed in the form of y = *A*/(1 + *be*^-ct^). The values of regression constants (a, b, and c) are in Table 4.

Fig 2 shows the trends of height, girth and total dry biomass of rubber plants in relation to different fertilizer treatments for immature rubber grown on two soil series over the duration of the experiment. As indicated in the plotted graph, the parameters under consideration i.e. height, girth and total dry biomass were well fitted to the logistic growth model expressed as $y=\frac{A}{1+be^{-ct}}$ where y = height/stem girth/ dry biomass, t = months after transplanting and A, b and c were constants (Table 4). Choosing an appropriate model depends on many factors including the relative stability of the model to describe the studied parameter i.e., growth and the low residual standard deviation. The data on rubber growth have been tested with different growth models, the retained model characterizes the vegetative growth of the rubber plant in relation to the results of Table 4. The plant performance of plants in different soils under different treatments illustrated in Fig 2 denoted that the growth trend is adequately described by the growth function of y = A/(1+be−ct) but with different growth rates.

**Table 4:** Constant (a, b and c), R2, F value, P≥F of plant height, girth and plant dry biomass (y) versus months after planting (MAP = t) with the function of $\left(lny=\frac{A}{1+be^{-ct}}\right)$.

Variable	Soil Type	Trt	a	b	c	R^2^	F-value	p≥F
Plant height	Bungor	1	1	-0.4609	-0.6614	0.98	3177.13	< .0001
		2	1	-0.4999	-0.8032	0.99	3485.64	< .0001
		3	1	-0.4313	-0.6526	0.99	2519.84	< .0001
		4	1	-0.457	-0.7181	0.99	25050.5	< .0001
		5	1	-0.4619	-0.6727	0.99	2636.61	< .0001
	Segamat	1	1	-0.4717	-0.6961	0.99	2566.85	< .0001
		2	1	-0.4953	-0.7899	0.99	6143.2	< .0001
		3	1	-0.4573	-0.7095	0.99	4019.75	< .0001
		4	1	-0.4732	-0.7413	0.99	17676.6	< .0001
		5	1	-0.468	-0.6974	0.99	17676.6	< .0001
Stem Girth	Bungor	1	1	-0.5185	-0.8458	0.99	6261.28	< .0001
		2	1	-0.5441	-0.8731	0.99	2662.21	< .0001
		3	1	-0.5405	-0.8662	0.99	6587.67	< .0001
		4	1	-0.5502	-0.8266	0.99	15273.7	< .0001
		5	1	-0.3705	-0.9213	0.99	15198.5	< .0001
	Segamat	1	1	-0.5378	-0.8413	0.99	6263.46	< .0001
		2	1	-0.4848	-0.9061	0.99	5489.95	< .0001
		3	1	-0.5511	-0.8322	0.99	10393.8	< .0001
		4	1	-0.5202	-0.8913	0.99	7790.89	< .0001
		5	1	-0.5434	-0.8694	0.99	7019.79	< .0001
Plant Biomass	Bungor	1	1	-0.5104	-0.7859	0.99	5199.43	< .0001
		2	1	-0.554	-0.7819	0.99	4171.36	< .0001
		3	1	-0.5179	-0.7553	0.99	16952.2	< .0001
		4	1	-0.544	-0.7503	0.99	2301.38	< .0001
		5	1	0.4827	-0.7076	0.99	20555.7	< .0001
	Segamat	1	1	-0.5242	-0.8193	0.99	22927.8	< .0001
		2	1	-0.497	-0.7455	0.99	6310.86	< .0001
		3	1	-0.4977	-0.7686	0.99	3287.32	< .0001
		4	1	-0.5257	-0.7958	0.99	24271.9	< .0001
		5	1	-0.504	-0.7765	0.99	7336.98	< .0001

The quadratic model of this exponential function is the best model to express the growth dynamics for the respective parameters compared to simple linear and cubic models. The R^2^ values were very high in the range of 0.98–0.99 with p < 0.01. The respective regression constant values of the equation are indicated in Table 4. The dynamics or trends of height, girth, and total dry matter accumulations for all plants grown on the two soil types were similar in the period of evaluation. The function of height, stem girth and plant biomass versus months after transplanting for each fertilizer treatment applied had a high F-value level at a = 0.0001 (Table 4). This indicates that the growth curve was significantly associated with each treatment.

Direct measurement from time to time is one of the classic ways to measure growth. Growth data can be regressed with time to study the trend of growth in relation to treatments applied. In the early stage, the growth increased steadily over time across all fertilizer treatments applied to the rubber seedlings. Based on this model, it can be deduced that there was a positive response of the plants to treatments increased. This observation was supported by the previous modelling of the vegetative growth curve in *Hevea brasiliensis* [19] where a linear growth response was recorded in the vegetative growth. The rapid linear growing phase is usually followed by a consequent period of slow growth showing a concave tendency. The growth will then continue by an absolute linear form, forming a sigmoid curve.

The plotted graph indicated that the growth of *Hevea brasiliensis* was properly fitted into a logistic growth model expressed as y = A/(1+be^-ct^), where y = stem girth/plant height, t = months after transplanting and A, b and c were constants as indicated in Table 4. Previous studies on the changes in vegetative growth of *Hevea brasiliensis* by Obouayeba *et al*. [15] and Debouche [32] established that high values were recorded in different clones (PB 235, GT 1 and PR 107) during the early growth stages of the rubber plant up to up to three and half years, with the highest increase recorded at the end of this period. In other words, similar species of *Hevea brasiliensis* were adequately represented by the same growth model. The growth of plants is a continual function, however, in all plants, there is a maximum value of M, not attained but approached by size when the time is indefinitely prolonged [15]. This function is limited by the M value. As growth is an irreversible increase, the function does not decrease. As girth is always positive, negative values are described in the function. Considering the above factors, the choice of model to be selected is limited to those continuous, without negative, decreasing and limited functions.

In the present research, the growth curve will most likely become stagnant if the duration of the experiment is extended. The explanation for this is that the experiment was conducted in a limited soil volume. The rubber plant is a dicotyledon, and thus requires deep soil for adequate and healthy root establishment [33, 34]. Recent studies have shown the influence of soil volume on the growth performance of *Hevea brasiliensis* where limited soil volume enormously restricted the growth of rubber plants in terms of dry matter production and root properties such as root length, size and volume [35].

**Fig 3:**
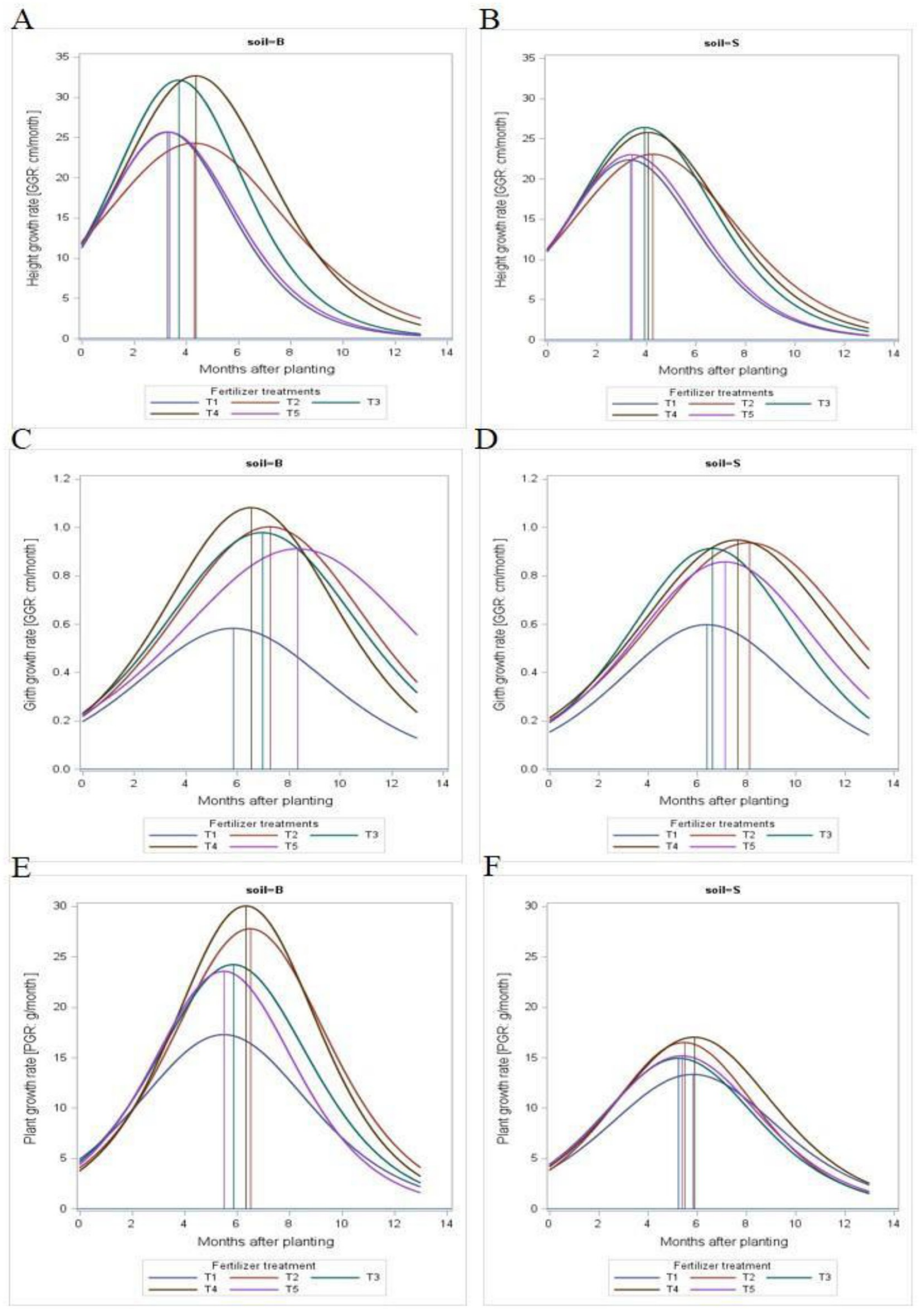
Height growth rate (HGR), girth growth rate (GGR), rubber growth dynamics i.e. dry biomass in the two on the two studied soils (B-Ultisol and S-Oxisol) expressed in the form of $\frac{dy}{dx}=\frac{Abce^{-ct}}{(1+be^{-ct)\;z}}$ for the study period, where a, b and c are constants as indicated in Table 4.

Dynamics in the growth of *Hevea brasiliensis* could be examined by modelling the growth pattern using an instantaneous method. This is conducted by computing the derivatives of the equation related to the Fig 2 and also the constants in the table, the height growth rate, stem growth rate and biomass growth rate at current (instantaneous) versus month after transplanting [dy/dx = Abce^-ct^/(1+be^-ct^)^2^ where y, t, A, b and c are as described above] were shown in Fig 3.

The growth rate of height (GR_H_), girth (GR_G_) and total dry biomass (GR_B_) of immature rubber cultivated on the soils are computed using the mean method. Fig 3 shows the result of the growth rate computation. The growth rates for all parameters were similar in the two studied soils. The height growth rate was on an upward trajectory and rapid increase in height were recorded in all treatments in the two soils series showing remarkable growth of the young seedlings in both the Ultisol and Oxisol up to the point of inflection occurring at the later part of the 3^rd^ month after transplanting to early period of the 4^th^ month. The remarkable growth rate for plant height may be attributed to plant growth in response to light.

In terms of girth growth rate (GR_G_) for rubber seedlings grown on the two studied soils, the girth consistently increased from the period of transplanting in both the Ultisol and Oxisol up to the point of inflection which varies from around 6-month to the middle of the 8^th^ month in the Ultisol and middle of 6 month to the early part of 8^th^ month in the Oxisol. As shown in Fig 3, it can be observed that maximum girth growth occurred in treatments that received MRSG at the recommended rate (T4) while the lowest growth occurred in control (T1).

The data on plant growth rate in the two studied soils as indicated by the total dry biomass is presented in Fig 3. It was observed that plant growth rate increased consistently from the period of transplanting up to the point of inflection where the maximum rate of increase in plant growth rate was recorded. The point of inflection ranged from the later period of the fifth month up to the middle part of the 6^th^ month in the Ultisol while inflection period was recorded in the later period of the fifth month up to the early part of the 6^th^ month in the Oxisol. The Figure shows that maximum girth growth occurred in treatments that received MRSG at the recommended rate (T4) while the lowest growth occurred in control (T1).

**Fig 4:**
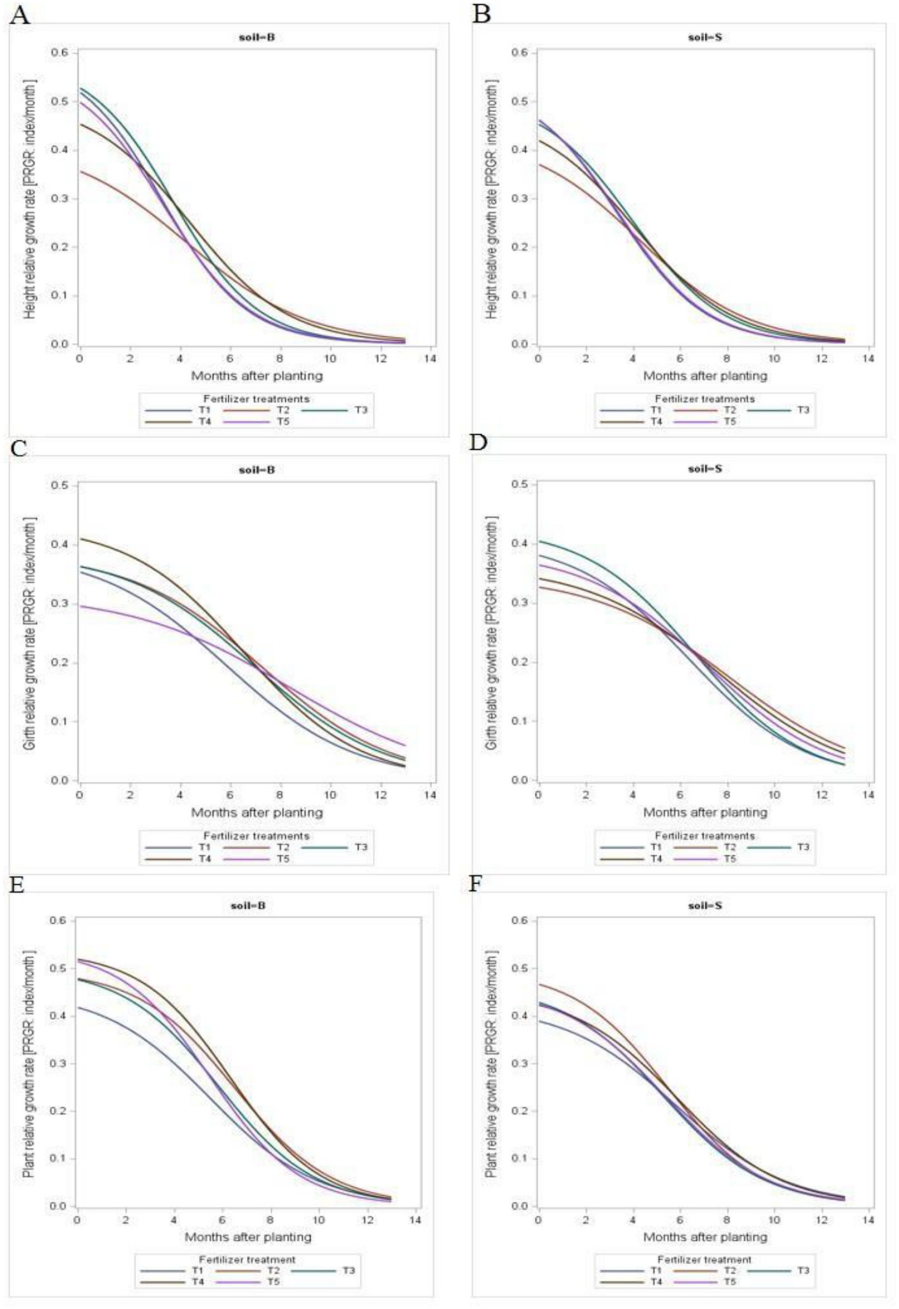
Relative growth rate (RGR) in terms of plant height, stem girth and total dry biomass for rubber grown on two soil series (Ultisol and Oxisol) in relation to different fertilizer types for the growth period.

Fig 4 shows the relative growth rate of *Hevea brasiliensis* computed using the instantaneous method. In simple words, the relative growth rate (RGR) in terms of height, stem girth and total plant biomass is the ratio of the growth rate (height or stem girth or total plant biomass) to the current (instantaneous) height or stem girth or total plant biomass of the plants at a specific time. For RGR_H_, GML treatment had the highest value at 12 months of transplanting for plant grown in the Bungor soil (Ultisol) and this was followed by MRSG treatment at the recommended rate (Fig 4). The same trend was also recorded in the Segamat soil (Oxisol) however, at planting, Treatment (T3) had the RGR_H_ in the Ultisol while MRSG at double the recommended rate had the highest value for RGR_H_ in the Oxisol. The RGR_G_, in terms of stem girth (Fig 4) exponentially decreased from 0.30–0.42 and 0.32–0.41 month (at 0 month after transplanting) to 0.03–0.05 and 0.03–0.04 month‴ (at 12 months after transplanting) for plants grown on different fertilizer treatments on the Ultisol and Oxisol, respectively.

The relative growth rate would be more appropriate to assess overall plant growth for the whole study period. Relative growth rate (RGR) expresses the rate of gain in plant height, girth or plant biomass per unit time. Higher growth in the early period of study could be related to plant response to treatment and different environments, from a small container to a larger polybag. Long term study (7 years) on the growth of *Hevea brasiliensis* [36] showed that RGR for RRIM 501 and RRIM 513 clones of rubber plant increased initially after bud grafting, whereas a consistent decline was recorded thereafter. The researcher further noted that a steady decline in the relative growth rate was attributed to the continuous decline in the net assimilation rate. As a result, there was a decrease in both the net assimilation rate and leaf area ratio. The reduction in leaf area was attributed to the increasing ratio of total plant weight which comprises non-photosynthetic tissues. Biomass allometry such as the production of new leaf and other non-photosynthetic tissues such as branch will decrease RGR [36]. Branching will provide basic framework for an increased assimilatory surface. The plant will develop more leaves in order to increase photosynthesis capacity during the early period after transplanting [20].

### 3.2 Growth calibration curve and correlation study

The growth of a plant can be expressed in terms of biomass production [19]. Across all treatments, total biomass was higher in magnesium fertilizer-treated soils compared to untreated ones. Meanwhile, MRSG treatments had a comparatively higher mean value for biomass in the two soils across all months, indicating that MRSG application led to a notable increase in plant growth. The total dry weight of rubber seedlings was plotted against Mg level in the soil, where it was noted that a significant relationship exists between biomass and the level of exchangeable magnesium in the Ultisol (R^2^ value of 0.59). Meanwhile, no significant relationship was recorded between the level of Mg level and plant biomass on the Oxisol (R^2^ value of 0.41). However, when biomass was measured against soil calcium level, a significant relationship was recorded in both soils with R^2^ = 0.96 and 0.79, respectively and the relationship between the variables can be expressed as = 132.53+50.23+ 132.53+50.22. This suggested that Ca was more influential towards predicting the growth of rubber seedlings in the study. The result agrees with similar research where MRSG applied as Mg fertilizer was tested on oil palm seedlings in the glasshouse and on the field and a positive effect of soil nutrients was observed on growth indicators. In the research of Ayanda *et al*. [25], it was observed that Ca was more limiting in the soil and better correlated with oil palm growth compared to exchangeable Mg. The remarkable growth of the plant may be attributed to the role of Ca in cell wall division and root growth, meanwhile, other authors have reported that plant growth will proceed optimally when the root is adequately developed [33, 37].

Similarly, soil pH significantly influences the growth of rubber seedlings. Data from this study indicated that the growth of rubber seedlings expressed by biomass production showed a significant positive linear relationship between soil pH and total dry biomass. This was consistent with a previous study conducted on rubber seedlings by Shafar [19], where a positive linear relationship was reported between pH and plant biomass. When the pH of the soil solution is above 5, root growth is unaffected by aluminium toxicity, which is a characteristic of acid soils that hinders root proliferation. At this pH (>5) nutrients in the soil become available for plant uptake. The result of the present trial indicated there is a positive linear relationship between pH, exchangeable Ca and Mg in the soil.

A multiple regression analysis of soil pH, magnesium and calcium indicated the existence of a significant relationship between these variables and the biomass of rubber plants growing in the glasshouse. The R-Square of 0.825 was obtained in the regression analysis (i.e. 82.5% of the relationship is described by the regression line). The intercept (166.66) and the regression coefficient of soil pH (11.27), exchangeable calcium (46.19) and exchangeable magnesium (49.88) of this relationship, represented by y = 166.66+11.27+46.19+49.88 were not significant. However, using the stepwise regression method, it was determined that exchangeable Ca was the variable that contributed most significantly to the biomass growth of rubber seedlings. The result showed that the relationship between biomass and exchangeable Ca is significant (Pr>F = 0.04) with R-square = 0.79. The intercept (132.53) and regression coefficient (50.23) are significant and the relationship between biomass and exchangeable Ca is written as Biomass = 132.53+50.22. The result also established soil pH is positively correlated with exchangeable Ca. Thus, there is the assumption that if soil pH, exchangeable Mg and Ca increases, the growth of rubber seedlings will increase significantly. It is known that at pH above 5, exchangeable in soil solution Al is precipitated as inert Al hydroxide. Thus, soil nutrient imbalance, nutrient unavailability and other limitations from soil acidity will be corrected, thereby contributing to improve the growth of plants in the soil.

The relative biomass (%) values were determined and plotted against Ca to find the critical calcium level required to support rubber growth. The critical exchangeable calcium value predicted through this method was 1.05 cmol_c_kg^-1^ and 0.78 cmol_c_kg^-1^ (value measured at 90% relative plant height) in the Ultisol and Oxisol, respectively. These levels of exchangeable Ca are uncommon in acid soils under continuous cultivation in Malaysia, therefore the level of exchangeable Ca should be increased via nutrient management. This study showed that at this level of exchangeable calcium, soil pH goes toward 5 as a result acidity no longer becomes a problem to the plant and nutrients becomes available for plant uptake. The result of this study supports previous observation by Ayanda et al. [13] that MRSG contains a high amount of secondary nutrients and further showed that MRSG can supply the secondary nutrients (Ca and Mg) required to sustain rubber growth, giving better or similar performance to conventional fertilizers i.e. GML and kieserite in terms of crop growth improvement. This notion was supported by research findings on the improvement of soil condition, matured and immature oil palm growth using MRSG as a nutrient source and acid soil ameliorant [25].

## 4.0 Conclusion

The result of the reported plant growth experiment indicated the growth of rubber seedlings in the tested soils was improved by the increasing level of Ca and Mg due to MRSG treatment. The untreated soils Ca and Mg levels were less than the critical level required to support the healthy growth of rubber seedlings in the Ultisol and Oxisol tested. The challenge of inherent nutrient deficiency in the soil was overcome through MRSG application in the two studied soils. The current research confirmed that MRSG gives a comparable level of Mg similar to kieserite. MRSG is also a source of exchangeable Ca which increases the soil pH and improves plant growth; therefore, MRSG aside its Mg and Ca properties; has liming properties and serves as an important soil ameliorant similar to GML in limiting the Al in soil solution and raising soil pH levels.

From this study, the results indicated that MRSG application to acid soils, Ultisol and Oxisol significantly influenced the growth and nutrition of young rubber. According to the results obtained, the application of MRSG significantly influenced the fertility of the soil and the growth of *Hevea brasiliensis* at the immature stage. MRSG had a noticeable impact on rubber growth after three months and increased throughout the duration of the study. This research expressed the logistic growth pattern of rubber plant (heigh, girth and total dry biomass) vs months as a model in the form of y = A/(1+be-ct). This model was found to be ideal for describing the dynamics of rubber growth in terms of the studied parameters in relation to the months after transplanting.
